# Treatment of Anemia in Transfusion-Dependent and Non-Transfusion-Dependent Lower-Risk MDS: Current and Emerging Strategies

**DOI:** 10.1097/HS9.0000000000000314

**Published:** 2019-10-30

**Authors:** Ulrich Germing, Ester N. Oliva, Devendra Hiwase, Antonio Almeida

**Affiliations:** 1 Department of Hematology, Oncology and Clinical Immunology, Universitätsklinikum Düsseldorf, Düsseldorf, Germany; 2 Department of Hematology, Grande Ospedale Metropolitano Bianchi Melacrino Morelli, Reggio Calabria, Italy; 3 Hematology, Royal Adelaide Hospital, Adelaide, SA, Australia; 4 Clinical Hematology, Hospital da Luz Lisboa, Lisbon, Portugal.

## Abstract

Myelodysplastic syndromes (MDS) are a heterogeneous group of bone marrow disorders with a highly diverse clinical course. For lower-risk MDS patients, therapeutic objectives aim to correct chronic anemia and improve/maintain health-related quality of life (HRQoL). However, disease burden is often insufficiently recognized, and although some patients do not respond/lose response to standard treatment, many are treated late. This is the case for non-transfusion-dependent patients with symptomatic anemia, in whom delayed treatment initiation may lead to unnecessary morbidity. Current active treatment options for lower-risk MDS are limited. Standard care for lower-risk 5q deletion [del(5q)] MDS patients with anemia remains supportive, consisting of red blood cell (RBC) transfusions, iron chelation therapy, and treatment with erythropoiesis-stimulating agents (ESAs) in the case of low serum erythropoietin levels. Response rates to ESAs range from 15% to 63%, whereas 56% to 67% of patients with del(5q) MDS achieve RBC transfusion independence with lenalidomide. Treatment options for patients’ refractory to ESAs and/or lenalidomide, however, are limited. Frequent transfusions are associated with profound clinical, HRQoL, and economic consequences for transfusion-dependent patients. This review focuses on the multiple unmet clinical needs that exist in the treatment of anemia associated with lower-risk MDS and the current and future treatment options that may improve disease management and patient outcomes.

## Introduction

Myelodysplastic syndromes (MDS) constitute a heterogeneous group of bone marrow disorders characterized by ineffective hematopoiesis resulting in peripheral cytopenias, especially anemia, and a risk of progression to acute myeloid leukemia (AML).^1,2^ The incidence of MDS is 4.15/100,000 annually in the European Union (EU);^3^ in the United States (US), the age-adjusted annual incidence was 4.9/100,000 in 2007 to 2011 and the median age at diagnosis is approximately 70 years.^4^ The prevalence of MDS in the EU is 7/100,000 people^3^ and in the US its current estimate of 60,000 to 170,000 patients is projected to grow as the average age of the population increases.^4^

Because MDS takes a highly heterogeneous clinical course, several prognostic scoring systems have been developed to facilitate therapeutic decision-making based on patients’ risk profiles. The International Prognostic Scoring System (IPSS), a risk assessment tool for patients with de novo MDS, classifies patients on the basis of karyotype, number of cytopenias, and percentage of bone marrow blasts into four prognostic groups: Low, Intermediate-1 (Int-1), Intermediate-2 (Int-2), and High risk.^5^ These IPSS categories are often grouped into “lower-risk” MDS (ie, IPSS Low and Int-1 risk) and “higher-risk” MDS (ie, IPSS Int-2 and High risk), and dictate the recommended course of treatment. Risk-group definitions have been further refined in the recently revised IPSS (IPSS-R),^6^ which stratifies patients into five risk categories (Very low, Low, Intermediate, High, and Very high risk) that take into account the degree, rather than the presence of, cytopenias and new chromosomal and blast categories. These categories are associated with clear differences in overall survival and progression to AML and the IPSS-R has been validated in several independent studies.^7–9^ Most patients with lower-risk MDS, as defined by IPSS categories, fall into the IPSS-R Very low-, Low-, or Intermediate-risk categories and account for approximately three-quarters of patients with MDS, representing a prevalence of approximately 5.25/100,000 Europeans.^3,6^ These patients generally have a better prognosis than patients in the higher-risk groups, with median overall survival ranging from 3.0 to 8.8 years vs 0.8 to 1.6 years, respectively.^6^ The IPSS-R is a superior risk stratification system compared to the original IPSS for predicting both disease progression and survival. During validation of the IPSS-R, approximately 7% of patients in the Int-1 IPSS category were shown to have either High or Very high risk according to the IPSS-R.^9^ Due to the changing nature of risk over time, researchers have suggested the IPSS-R High risk category should be raised to ≥3.5 points from ≥3 points.^10^ Further studies will likely help clarify recommendations in this dynamic research area. We recommend that, in future studies, “lower-risk” patients are defined according to the IPSS-R Very low-, Low-, and Intermediate-risk categories. Due to the nature of current guidelines and available publications however, the data discussed herein will often refer to the previous IPSS definition.

Identification of non-transfusion dependent (NTD) patients who are at an increased risk of disease progression may be further facilitated by the use of mutational analysis.^11,12^ Combining *EZH2* mutation status with the new MD Anderson Lower-Risk Prognostic Scoring System (LR-MDSS), particularly applicable to Low-risk and Int-1 MDS, identified 29% of patients with lower-risk MDS who had a worse-than-expected prognosis.^13^*TP53* mutations occur in 19% of patients with MDS with isolated chromosome 5q deletion [del(5q)] and are correlated with leukemic progression.^14^ The adverse impact of *TP53* persists after adjustment for cytogenetic risk and is of practical importance in evaluating prognosis. Nevertheless, there remains a crucial need for specific biomarkers to allow early diagnosis and identification of patients with MDS who are at risk of becoming transfusion dependent (TD).

In healthy individuals, platelet counts range from 150 × 10^9^/L to 450 × 10^9^/L. Thrombocytopenia, defined as a platelet count <100 × 10^9^/L, is the cause of hemorrhagic complications in patients with MDS and is associated with shorter survival and increased risk of progression to AML.^15^ Severe thrombocytopenia has been shown to be an independent prognostic factor^16^ and the severity of thrombocytopenia has been included in the IPSS-R.^6^

For patients with lower-risk MDS, who are the focus of this review, the main therapeutic objectives are to correct chronic anemia and thrombocytopenia, reduce recurrent infections, and improve or maintain health-related quality of life (HRQoL).^17,18^ The ultimate treatment goal is to alter the natural course of the disease and to improve overall and disease-free survival. However, few treatment options exist. Standard care for patients with lower-risk MDS remains treatment with erythropoiesis-stimulating agents (ESAs), with granulocyte-colony stimulating factor (G-CSF) in patients with neutropenia and recurrent infections, cytomegalovirus-safe red blood cell (RBC) transfusions/G-CSF with erythropoietin (EPO) in the case of anemia,^17,19^ and supportive care with platelet transfusions in the presence of thrombocytopenia.^20^ During the course of their disease, 50% of patients with MDS will need RBC transfusions^9^ and 6% to 33% will need platelet transfusions.^21^

The main parameters for consideration when planning therapeutic interventions for patients with lower-risk MDS are cell counts, ferritin and endogenous EPO levels, the presence of del(5q), and, most important, the burden of disease as reflected by general health status and symptoms of hematopoietic insufficiency. Hematopoietic insufficiency primarily refers to symptoms of anemia and, less frequently, to thrombocytopenia. While there is correlation between hemoglobin (Hb) levels and the presence and significance of clinical symptoms of anemia, there is wide variability with regards to Hb levels that trigger the need for transfusion or initiation of treatment. These parameters, and particularly the term “symptomatic cytopenia”, are the basis of treatment algorithms in both the European LeukemiaNet (ELN)^2^ and National Comprehensive Cancer Network (NCCN) guidelines.^17^ If a symptomatic cytopenia becomes evident, clinicians then consider ferritin levels indicating iron toxicity, low serum EPO levels indicating the potential use of ESA, and the presence of del(5q) indicating the option to treat with lenalidomide. In contrast, a low EPO level and the presence of del(5q) without symptoms of cytopenia would never prompt a clinician to start any treatment.

Transfusion dependence is considered a negative prognostic factor and overall survival is shorter for TD patients than for NTD patients.^22,23^ The concept of transfusion dependence continues to be a topic of fierce debate. Patients requiring ≥2 units of RBCs per 28 days have commonly been considered TD.^24–26^ More recently, an International Working Group (IWG) response paper split TD patients into two groups of high (≥8 RBC transfusions/16 weeks) and low (3–7 RBC transfusions/16 weeks) transfusion intensity.^27^ In addition, analysis of the large European MDS Registry showed that a transfusion dose density of <3 units was associated with an increased risk of progression.^28^ Therefore, when considering risk, it may be informative to consider all patients receiving regular transfusions as TD.

Multiple unmet clinical needs exist in the treatment of anemia in patients with lower-risk MDS, whether they are TD or NTD. Those unmet needs, along with current and future treatment options that may improve disease management and outcomes for patients with lower-risk MDS, are the focus of this review.

## Treatment of anemia

Fatigue is the primary symptom of anemia. Physicians managing patients with MDS should be aware of signs of fatigue, alongside other symptoms such as lethargy, dizziness, shortness of breath, headache, and heart palpitations.^29^ These clinical signposts are complicated in the oncology environment by the overlap with effects of both the underlying disease and treatment. Assessment of the presence of symptomatic anemia is primarily based on patients reported medical needs. In our experience, we allow the patient to decide to commence treatment if his or her quality of life is worsened significantly by low cell counts, while taking into account patient characteristics such as age and co-morbidities.

**Figure 1:**
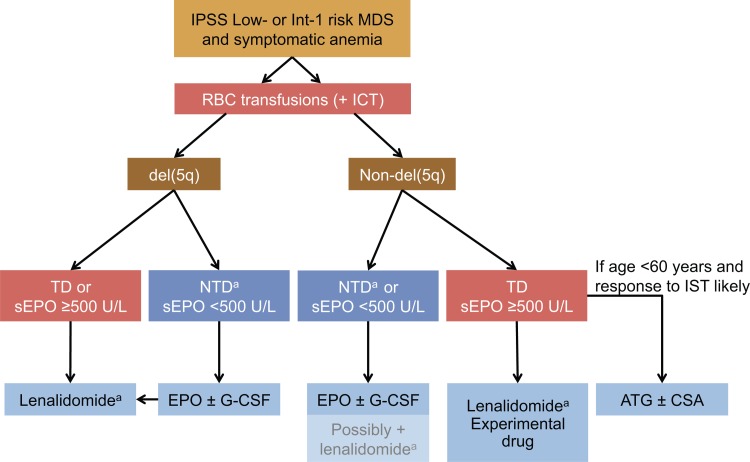
**Guidelines from the NCCN, ESMO, and ELN for lower-risk MDS patients**. Transfusion dependence is defined as an RBC transfusion need of ≥2 units/month. ATG = antithymocyte globulin; CSA = cyclosporin A; del(5q) = chromosome 5q deletion; ELN = European LeukemiaNet; EPO = erythropoietin; ESMO = European Society for Medical Oncology; G-CSF = granulocyte-colony stimulating factor; ICT = iron chelation therapy; Int = Intermediate; IPSS = International Prognostic Scoring System; IST = immunosuppressive therapy; MDS = myelodysplastic syndromes; NCCN = National Comprehensive Cancer Network; NTD = non-transfusion dependent; RBC = red blood cell; sEPO = serum erythropoietin; TD = transfusion dependent. ^a^ Lenalidomide is not licensed for the treatment of lower-risk MDS in the EU. Hypomethylating agents may be considered in special cases (approved in the USA only).

Recommendations for the management of anemia in patients with lower-risk MDS are provided in guidelines prepared by the NCCN,^17^ the European Society for Medical Oncology (ESMO),^19^ and the ELN group.^2^ Those guidelines have broadly similar recommendations for the treatment of symptomatic anemia, as summarized in Figure 1. The initial treatment decision is based on the degree of anemia (ie, whether the patient is symptomatic), the presence or absence of del(5q), and serum EPO levels. Before initiating treatment for MDS-related anemia, however, it is important to rule out or identify and treat any co-existing causes of anemia, such as gastrointestinal bleeding, hemolysis, renal disease, or nutritional deficiencies.^17^

Recommended first-line active treatment options for MDS-related, symptomatic anemia include ESAs, specifically epoetin alfa at a starting dose of 30,000 to 60,000 IU with a target Hb range of 10 to 12 mg/dL (not exceeding 12 mg/dL) and dose decrease to tolerance for patients with serum EPO ≤500 mU/mL. In non-responders, G-CSF is recommended after 8 weeks at 300 μg in 2 to 3 divided doses.^2,17^ Lenalidomide is recommended for patients with del(5q). Immunosuppressive therapy (IST) is a viable option for younger patients with serum EPO >500 mU/mL and features favorable for a response to IST. RBC transfusions, in addition to active treatment, remain important for all patients with symptomatic anemia, as this may be the only option for those who do not respond or who are not eligible for any of the active therapies described above. It is important to note that RBC transfusions are only recommended for patients with moderate to severe anemia, defined as a Hb concentration <10 g/dL, or symptomatic anemia.^2,17,19^

Reduced erythropoiesis is a risk factor for iron toxicity in all MDS patients, regardless of transfusion requirement, due to the fact that impaired erythropoiesis reduces levels of hepcidin which leads to increased iron uptake by the liver.^30^ The major risk factor for iron toxicity in MDS patients, however, is frequent RBC transfusions. Complications related to iron toxicity include liver damage and cardiomyopathy. Furthermore, increased serum ferritin levels are associated with increased mortality independent of transfusion.^30,31^

To reduce iron accumulation, iron chelation therapy (ICT) is often required. The decision to initiate ICT should be based on the patient's risk of iron toxicity-related complications. The impact of transfusional iron toxicity and when to initiate ICT is less clearly defined.^32^ European guidelines suggest considering ICT in TD patients with isolated del(5q) and a serum ferritin level higher than 1000 ng/mL after approximately 25 units of RBCs or as conditioning in patients who are candidates for allogeneic stem cell transplantation (allo-SCT).^2^ In contrast, the NCCN Guidelines recommend evaluation and ICT if >20–30 RBC transfusions have been received.^17^ Emerging evidence may point to a wider therapeutic role for ICT, as it has also been shown to improve hematopoiesis, reduce RBC transfusion requirements, and improve overall survival in a proportion of patients with lower-risk MDS.^30,33^ In spite of these potential benefits, ICT is not without risk. Adverse events include injection-site reactions and gastrointestinal disturbances. Rare but severe events such as renal failure have also been reported. The success of ICT also depends strongly on patient adherence.^32^

## Key clinical challenges for TD patients

Frequent RBC transfusions are associated with profound clinical, HRQoL, and economic consequences for TD patients.^34,35^ In addition to the aforementioned complications related to iron toxicity, alloimmunization, allergic reactions, and transfusion-transmitted infections can also occur.^36–39^ Dependence on RBC transfusions may also have a negative impact on survival. In a retrospective analysis of 467 patients with MDS, transfusion dependence was associated with both shorter overall survival (hazard ratio [HR] = 2.16; p < 0.001) and shorter leukemia-free survival (HR = 2.02; p < 0.001).^23^ A study of 381 patients with lower-risk MDS and del(5q) showed that TD patients had significantly shorter overall survival than NTD patients (44 vs 97 months; p < 0.0001) and transfusion dependence at diagnosis was associated with an increased risk of progression to AML.^40^ In a meta-analysis of the association between overall survival and transfusion status in patients with MDS, the risk of death was 59% lower for NTD patients than for TD patients.^41^

Transfusion dependence has a significant impact on physical, functional, and social well-being and plays an independent and major role in the deterioration of HRQoL.^42–45^ It may also affect patients’ perception of MDS-related disturbances, such as dyspnea, dependence on hospital staff, and inability to travel.^43^ Chronic blood transfusions are also time-consuming for the patient and may pose a psychosocial burden on patients and their families.^34^ In one study, 34% of TD patients felt that blood transfusions were burdening their family and 65% reported that they would prefer a treatment that temporarily makes them feel worse if it could reduce or remove the need for transfusions.^46^ In lower-risk MDS, Hb concentration is an important predictor of HRQoL^43,47,48^ and a fluctuating Hb concentration, typically associated with RBC transfusions, may negatively affect HRQoL.^49^

In addition to the clinical and HRQoL burdens described above, TD patients face an economic burden. Long-term dependence on transfusions is associated with increased healthcare costs, especially for patients needing a higher transfusion frequency.^50^ A retrospective review of Medicare claims data showed that the 3-year cumulative mean cost per patient was substantially higher for TD patients than for NTD patients (USD 88,824 vs USD 29,519, respectively).^51^ Similar relations between transfusion status and cost of care have been observed in other studies.^42,50,52^

## Key clinical challenges for NTD patients with symptomatic anemia

The burden of disease in NTD patients may be more substantial than previously recognized. Anemia due to MDS is often insufficiently recognized and undertreated. This is partly because MDS is a disease of the elderly, in whom anemia due to MDS may be difficult to distinguish from other age-related or non-malignant causes of anemia.^53,54^ In a review of an electronic medical records database of treatment patterns in 5,162 patients with MDS, 59.7% had received no treatment for their MDS.^55^ A survey of 101 hematologists and oncologists revealed that 24% of patients with newly diagnosed lower-risk MDS received no immediate treatment and were monitored using a “watch and wait” approach.^21^

For patients not yet receiving transfusions, even mild anemia may substantially affect HRQoL. In a multivariate analysis of individuals aged 65 to 84 years, mild anemia (ie, Hb 10.0–11.9 g/dL in women and 10.0–12.9 g/dL in men) was independently associated with poorer selective attention, worse physical functioning, and fatigue when compared with no anemia.^56^ A more recent analysis of patients with lower-risk MDS and del(5q) showed that physical HRQoL was poor for 75% of NTD patients (n/N = 9/12) and Hb level was inversely correlated with QOL-E questionnaire scores for physical functioning (p = 0.035) and fatigue (p = 0.049).^57^ Anemia-related reductions in HRQoL are amenable to treatment and response to ESA therapy is a predictor of improved QoL in lower-risk MDS patients.^58^

The severity of anemia in NTD patients may also be a risk factor for early mortality. In a retrospective analysis of 840 patients with MDS, the degree of anemia was a significant predictor of cardiovascular death (p < 0.001), independent of transfusion status and IPSS risk.^59^ In NTD patients with lower-risk MDS and del(5q), 5-year overall survival rates were 65.4% for patients with Hb levels < 10 g/dL vs 81.6% for patients with Hb levels of ≥10.5 g/dL; however, this finding was not statistically significant.^57^

### Benefits associated with early, active treatment of symptomatic anemia

Early identification and treatment of symptomatic anemia may be the key to improving outcomes in patients with lower-risk MDS. Reduction or elimination of the need for transfusions reduces the risk of iron toxicity complications, and patients who receive active therapies have a lower risk of these complications than those who are TD. Evidence from two large, multicenter studies of lenalidomide in TD patients with del(5q) and lower-risk MDS suggests that a durable erythroid response to active treatment is associated with improved outcomes.^60,61^ Patients responding to lenalidomide had a prolonged overall survival and a longer time to progression to AML.^60,61^ Achievement of RBC transfusion independence (RBC-TI) was associated with a median overall survival of 4.3 years (vs 2.0 years in non-responders; p < 0.0001).^61^ Furthermore, patients achieving RBC-TI experienced a 47% reduction in relative risk of death and a 42% reduction in relative risk of progression to AML or death.^60^ Longer overall survival was also observed for lenalidomide responders in another trial in lower-risk MDS patients with del(5q).^62^ Lenalidomide is generally well tolerated, with the prevalence of discontinuations due to adverse events (AEs) approximately 10% to 15%. Primary AEs leading to discontinuation were neutropenia and thrombocytopenia; deep vein thrombosis was uncommon (4%).^60^

Data from prospective and retrospective studies suggest that a response to ESAs may also be associated with improved overall survival in patients with MDS.^1,63–65^ In a randomized phase III study of EPO with or without G-CSF in 110 TD patients with lower-risk MDS according to the French–American–British criteria, median overall survival was longer for patients with an erythroid response (5.5 years) than for those without an erythroid response (2.3 years).^63^ Treatment options for patients refractory to ESAs are limited and randomized trial data supporting the benefit of ESA therapy on progression to AML in patients with MDS are lacking.

For those patients who have an erythroid response to active treatment, there may be associated HRQoL benefits. In clinical trials in TD patients, response to lenalidomide (ie, achievement of RBC-TI and Hb increase) was associated with improved HRQoL in patients who had lower-risk MDS with^66,67^ or without^68^ del(5q). Similarly, a response to ESAs was associated with improved HRQoL.^63,69–71^

As patients gain long-term benefit from response to active treatment, it is key to identify those who are likely to respond and to initiate treatment at an appropriate time. Early initiation of active treatment may be associated with better outcomes in patients with MDS, whether they are TD or NTD. In a large (N = 897) non-interventional study using the ELN MDS (EUMDS) registry, ESA therapy significantly delayed the onset of transfusion dependence in NTD patients. Non-significant survival benefits were also evident.^72^ In a retrospective study (N = 543) conducted in Italy, endogenous EPO levels <50 mU/L were predictive of response in TD patients, while in NTD patients receipt of high-dose ESAs, abnormal creatinine levels, and endogenous EPO levels <50 mU/L predicted response. In this study, responders showed both a higher 5-year overall survival and leukemia-free survival.^73^ In a retrospective analysis of Medicare claims, a shorter time from onset of transfusion dependence to active therapy (ie, hypomethylating agents [HMAs] or lenalidomide) was associated with higher rates of RBC-TI in 508 patients with lower-risk MDS.^74^ Similar results were obtained in a retrospective database study of 610 TD patients, in whom a shorter time from transfusion dependence to initiation of ESA therapy was associated with a higher probability of achieving RBC-TI.^75^ In NTD patients, a shorter interval between diagnosis and onset of ESA therapy (<6 months) predicted a better response to ESA therapy.^76^ Early active treatment may prevent NTD patients from becoming TD, which, in turn, positively affects treatment outcome and HRQoL.

## Limitations and challenges of current therapies

Current options for the active treatment of lower-risk MDS are limited^35^ and vary among countries, resulting in differences in patient outcomes by geographic location.^2,17,19,77^ Those that are available or under evaluation in clinical trials are listed in Table 1.

**Table 1:**
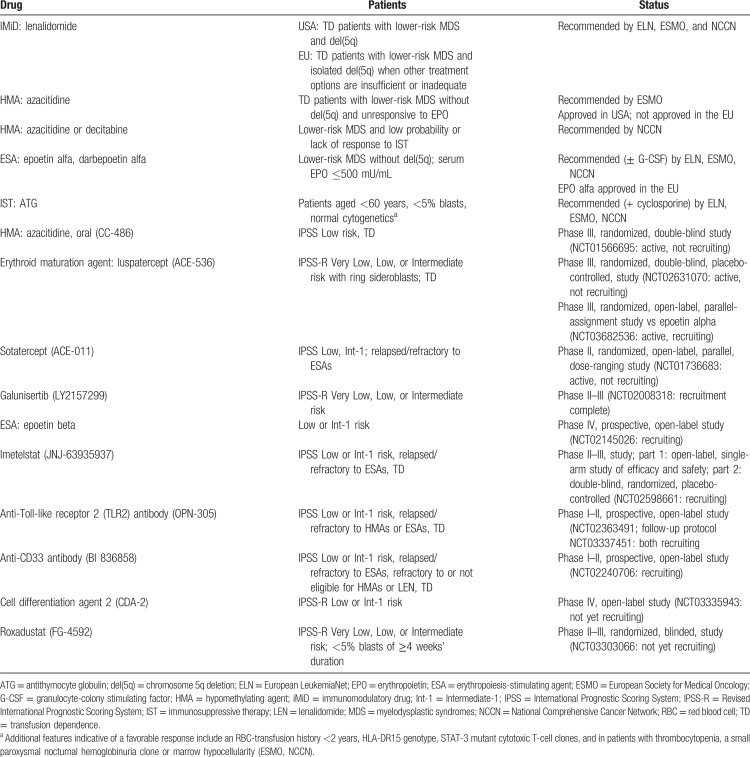
Active Therapies Recommended by the ELN, ESMO, or NCCN, or Under Study for the Treatment of Symptomatic Anemia in Patients with Lower-Risk MDS.

According to guidelines, ESAs, with or without G-CSF, are considered a first-line treatment for patients with lower-risk non-del(5q) MDS and a low transfusion burden (<2 RBC units/month) or with serum EPO ≤500 mU/mL.^2,17^ Epoetin alfa is approved in the EU for treatment of symptomatic anemia (Hb level ≤10 g/dL) in adults with Low- or Int-1 risk primary MDS who have low serum EPO (<200 mU/mL). Most responses to ESAs occur within 8 to 12 weeks of starting treatment.^2,78^ Rates of erythroid response to ESAs range from 15% to 63% in patients with anemia,^69,76,79^ but a durable response to ESAs is not achieved in all patients^69,76^ and early failure of an ESA is associated with a higher risk of progression to AML.^70,71^

Lenalidomide is approved in the US for the treatment of RBC-TD patients and lower-risk MDS with del(5q) and in the EU for the treatment of anemia in TD patients with lower-risk MDS associated with isolated del(5q) when other treatment options are insufficient or inadequate. In a phase III study in TD patients with lower-risk del(5q) MDS, treatment with 10 mg lenalidomide achieved RBC-TI ≥26 weeks in 56.1% of patients.^60^ A recent phase II trial reported achievement of transfusion independence by 67% of patients with lower-risk del(5q) MDS treated with lenalidomide.^62^

A treatment option for patients with lower-risk non-del(5q) MDS who have no response or stop responding to ESAs is a combination of lenalidomide and EPO with or without G-CSF.^17,80^ Interestingly, the response to lenalidomide in patients with lower-risk non-del(5q) MDS seems to be inversely correlated with the pre-treatment serum EPO levels.^81,82^ In particular, a randomized phase III study showed that patients with EPO ≤500 mU/mL had significantly higher response rates of RBC-TI ≥8 weeks vs patients with EPO >500 mU/mL (34.0% vs 15.5%; p = 0.015).^26^

IST provides another low-intensity, non-chemotherapeutic option. In an open-label, randomized, phase III trial, antithymocyte globulin (ATG) combined with cyclosporine resulted in a hematologic response in a subset of patients, but did not significantly improve transformation-free or overall survival rates.^83^ The NCCN recommends ATG with or without cyclosporine for patients who are aged 60 years or younger and have ≤5% marrow blasts, patients with marrow hypoplasia and paroxysmal nocturnal hemoglobinuria clone positivity, or STAT3 mutant cytotoxic T-cell clones.^17^ Although histocompatibility type HLA-DR15, mutations in *TP53*, *IDH1/2*, *ASXL1,* and *SF3B1,* and a paroxysmal nocturnal hemoglobinuria clone have been described as predictors of response, their predictive values were not confirmed in a large cohort of patients with MDS treated with IST.^84^

### Predictors of response to current therapies

Currently, there are few predictive markers for response that can guide treatment decisions for patients with lower-risk MDS, such as low endogenous EPO levels predicting response to ESAs or presence of del(5q) indicating lenalidomide treatment.^17,85^ Additional predictive molecular biomarkers would have great clinical utility and in recent years, much research has been focused on the identification of predictive gene mutations.^86^ However, currently there are no evident molecular biomarkers for response to ESAs. A study in 79 patients with lower-risk MDS found no statistically significant association between gene mutation profile and response to ESAs.^87^

### Novel therapies

Several promising novel therapies are under development for lower-risk MDS. Luspatercept is a recombinant fusion protein containing the modified extracellular domain of activin-receptor type IIB linked to an Fc fragment of human IgG1. Luspatercept binds to specific ligands of the transforming growth factor beta (TGF-β) superfamily and promotes late-stage erythropoiesis.^88^ In a recent open-label phase II study, luspatercept ameliorated anemia in patients with lower-risk MDS, in whom an IWG-defined hematologic erythroid improvement^27,89^ was achieved in 65% of those with a low transfusion burden (ie, Hb <4 RBC units every 8 weeks) and in 35% of those with a high transfusion burden (ie, Hb ≥4 RBC units every 8 weeks).^88^ Positive results were recently presented from the phase III MEDALIST trial (NCT02631070^90^) evaluating the safety and efficacy of luspatercept in patients with IPSS-R-defined Very low-, Low-, or Intermediate-risk MDS and ring sideroblasts. The primary endpoint of ≥8 weeks of RBC transfusion independence was achieved by 38% of patients treated with luspatercept compared with 13% of patients who received placebo. There was also a significant advantage for luspatercept in achievement of ≥12 weeks of transfusion independence.^90^

Results were recently published from a phase II study of sotatercept, an activin-receptor type IIA fusion protein that acts as a ligand trap to neutralize negative regulators of late-stage erythropoiesis. Almost half (49%) of the patients who were enrolled following lack of response to ESA therapy achieved the primary outcome of hematologic improvement–erythroid (IWG 2006 criteria). Sotatercept was well tolerated and serious AEs occurred in 23% of patients.^91^

Studies of an oral formulation of azacitidine (CC-486) have shown that extended doses may provide effective long-term treatment for patients with lower-risk MDS.^92,93^ In an open-label, multicenter trial, overall response (defined as complete or partial remission, RBC or platelet transfusion independence, or hematologic improvement) was achieved by 36% of patients receiving 14-day dosing and 41% of those who received 21-day dosing.^92^ CC-486 is being evaluated in TD patients with IPSS lower-risk MDS and thrombocytopenia in the phase III QUAZAR trial (NCT01566695).

Other agents in early development (Table 1) include the telomerase inhibitor imetelstat (NCT02598661),^94,95^ the hypoxia-inducible factor inhibitor roxadustat (NCT03303066), the anti-CD33 antibody (BI 836858; NCT02240706), an anti-Toll-like receptor 2 (TLR2) antibody,^96^ and galunisertib, a small-molecule TGF-β inhibitor.^97^ In the phase II/III IMerge trial, 34% of patients treated with imetelstat were transfusion independent for 8 weeks and 16% for 12 weeks.^95^ The humanized anti-TLR2 antibody OPN-305 showed acceptable tolerability in a phase I trial.^96^ In a phase II trial of galunisertib, 26% patients achieved a hematologic improvement (a continuous 8-week response with at least a 4-unit reduction in transfusion requirement from baseline or Hb increase by at least 1.5 g/dL).^97^

## Conclusions

Anemia is associated with substantial clinical, economic, and HRQoL consequences. In lower-risk MDS, reducing transfusion dependence and anemia-related symptoms are the main therapeutic goals. RBC transfusions are a lifesaving treatment that can improve anemia, but do not address its underlying cause. Active treatment is beneficial because it reduces transfusion burden, may improve long-term outcomes (including survival and HRQoL), and decreases healthcare-associated costs.

Identification and treatment of symptomatic, MDS-related anemia are the key to improving outcomes in patients with lower-risk MDS. Although evidence is as lacking, it is likely that early treatment will provide great benefits in health outcomes. The need for treatment of NTD anemia is often insufficiently recognized and anemia remains undertreated in a high proportion of patients. Although international guidelines exist for the management of anemia in TD patients, standard regimens for NTD patients are less well defined and better strategies are needed to identify candidates for treatment to optimize outcomes in terms of response, overall survival, and HRQoL.

Current treatment options for anemia are limited and each of the available treatment modalities has shortcomings. Use of ESAs is dependent on patients’ serum EPO level and not all patients have a durable response to ESAs. There is an unmet clinical need for improved therapies for patients unresponsive or refractory to first-line treatments and the development of new active agents, such as luspatercept, may improve the treatment landscape.
